# *Aerococcus urinae* and *Aerococcus sanguinicola*: Susceptibility Testing of 120 Isolates to Six Antimicrobial Agents Using Disk Diffusion (EUCAST), Etest, and Broth Microdilution Techniques

**DOI:** 10.2174/1874285801711010160

**Published:** 2017-09-21

**Authors:** Derya Carkaci, Xiaohui C. Nielsen, Kurt Fuursted, Robert Skov, Ole Skovgaard, Emilio P. Trallero, Reto Lienhard, Jenny Åhman, Erika Matuschek, Gunnar Kahlmeter, Jens J. Christensen

**Affiliations:** 1 Department of Clinical Microbiology, Slagelse Hospital, Slagelse, Denmark; 2 Department of Microbiology and Infection Control, Reference Laboratory Statens Serum Institut, Copenhagen, Denmark; 3 Department of Science and Environment, Roskilde University, Roskilde, Denmark; 4 Department of Microbiology, Hospital Universitario Donostia, San Sebastián, Spain; 5 ADMED Microbiologie, La Chaux-de-Fonds, Switzerland; 6 EUCAST Development Laboratory, Clinical Microbiology, Central Hospital, Växjö, Sweden; 7 Department of Clinical Medicine, University of Copenhagen, Copenhagen, Denmark

**Keywords:** Aerococcus urinae, *Aerococcus sanguinicola*, *Antimicrobial susceptibility testing*, Urinary tract infections, Disk diffusion, Etest, Broth microdilution

## Abstract

**Background:**

*Aerococcus urinae* and *Aerococcus sanguinicola* are relatively newcomers and emerging organisms in clinical and microbiological practice. Both species have worldwide been associated with urinary tract infections. More rarely cases of bacteremia/septicemia and infective endocarditis have been reported. Treatment options are therefore important. Just recently, European recommendations on susceptibility testing and interpretive criteria have been released.

**Objective:**

In this investigation 120 *A. urinae* and *A. sanguinicola* isolates were tested for susceptibility to six antimicrobial agents: Penicillin, cefotaxime, meropenem, vancomycin, linezolid, and rifampicin.

**Methods:**

Three susceptibility testing methods were used; disk diffusion according to The European Committee on Antimicrobial Susceptibility Testing (EUCAST) standardized disk diffusion methodology and MIC determination with Etest and broth microdilution (BMD). All testing was performed with EUCAST media for fastidious organisms.

**Results:**

Data obtained in this study were part of the background data for establishing EUCAST breakpoints. MIC values obtained by Etest and BMD were well correlated with disk diffusion results.

**Conclusion:**

All isolates were found susceptible to all six antimicrobial agents: penicillin, cefotaxime, meropenem, vancomycin, linezolid, and rifampicin.

## 1 INTRODUCTION

During the past one to two decades, the clinical relevance of various catalase-negative, Gram-positive cocci has been increasingly pointed out. Among these, *Aerococcus urinae* and *Aerococcus sanguinicola* are relatively newcomers and emerging organisms in clinical and microbiological practice [1-3]. Both species have worldwide been associated with urinary tract infections especially in elderly predisposed patients. A prevalence of *A. urinae* isolates in up to 4% of urine specimens examined has been reported [4]. Both species have also been isolated from blood from patients suffering from urogenic bacteremia/septicemia, in few cases with complicating infective endocarditis. Isolates of both species have furthermore casuistically been isolated from other foci too [1].

Isolates from both species are characterized by *Staphylococcus*-like morphology in Gram stains with growth characteristics resembling α-hemolytic streptococci on blood containing agar plates, a negative catalase reaction, a consistent antibiogram demonstrating susceptibility to β-lactams, and for *A*. *urinae* resistance to sulfonamides and aminoglycosides [5]. Recognition of the species may be harmed by their fastidious growth, which not seldom require supplementation with CO_2_ for optimal growth [5]. Diagnosing infections caused by both organisms are still missed in many laboratories around the world. However, with the increasing use of MALDI-TOF mass spectrometry for species identification, both species are diagnosed with increasing frequency [6].

Data on *in vitro* susceptibility of *A. urinae* isolates are accumulating [3, 4, 7-10]. The number of susceptibility reports on *A. sanguinicola* are relatively sparse compared to *A. urinae* [7, 11-14]. A lack of standardized susceptibility test methods and interpretive criteria for *Aerococcus* spp. have been problematic for clinical laboratories and clinicians [9]. Most publications have used the interpretive criteria for viridans group streptococci when evaluating the susceptibility. In general, both species exhibit susceptibility to penicillin and vancomycin and low level resistance towards aminoglycosides [10]. *A. urinae* is susceptible to nitrofurantoin [13].

A variety of test methods have been reported, and interpretive criteria for streptococci, staphylococci, and even enterococci have been applied [9]. In order to obtain comparable results it is desirable to develop a standardized methodology and interpretation when performing susceptibility testing on especially these two *Aerococcus* species. In the last revision from August 2016 of the US guideline from the Clinical and Laboratory Standards Institute (CLSI) on “Methods for antimicrobial dilution and disk susceptibility testing of infrequently isolated or fastidious bacteria” (M45) [15] a table on aerococci has been included.

In this study, 120 aerococcal isolates, *A. urinae* (*n* = 81) and *A. sanguinicola* (*n* = 39), of European origin were tested for susceptibility to six antimicrobial agents using the methodology recommended by The European Committee on Antimicrobial Susceptibility Testing (EUCAST). Data obtained in this study acted as part of the decision background for creating European clinical breakpoints for *A. urinae* and *A. sanguinicola* [16].

## 2 MATERIALS AND METHODS

### 2.1 Isolates

Eighty-one isolates of *A. urinae* and 39 isolates of *A. sanguinicola* of European origin were examined. Of the 81 isolates of *A. urinae*, 58 were from urine, 22 from blood, and one foot wound isolate; 41 isolates originated from Switzerland, 37 isolates from Denmark and two isolates from Spain. Of the 39 isolates of *A. sanguinicola,* 37 were from urine and 2 from blood; 29 isolates originated from Switzerland, 8 isolates from Denmark and one isolate from Spain. Two type strains were also included: *A. urinae* CCUG 36881*^T^* and *A. sanguinicola* CCUG 43001*^T^*. All isolates were species identified using MALDI-TOF mass spectrometry (Bruker Daltonics, Germany).

### 2.2 Susceptibility Testing

Three antimicrobial susceptibility testing methods were used; disk diffusion according to EUCAST standardized disk diffusion methodology for fastidious organisms [17] and MIC determination using Etest (bioMérieux, France) and broth microdilution (BMD) according to ISO standard 20776-1 [18] using Thermo Scientific Sensititre panels (East Grinstead, UK). The following six antimicrobial agents were tested: Benzylpenicillin, cefotaxime, meropenem, vancomycin, linezolid, and rifampicin. Rifampicin was not included in the BMD panel, therefore only tested with disk diffusion and Etest. The following disks (Oxoid, UK) was used: Benzylpenicillin 1 unit, cefotaxime 5 µg, meropenem 10 µg, vancomycin 5 µg, linezolid 10 µg, and rifampicin 5 µg. The following Etest strips were used: Benzylpenicillin 0.002-32 mg/L, cefotaxime 0.016-256 mg/L, meropenem 0.002-32 mg/L, vancomycin 0.016-256 mg/L, linezolid 0.016-256 mg/L, and rifampicin 0.002-32 mg/L. BMD (Sensititre): Benzylpenicillin 0.03-4 mg/L, cefotaxime 0.12-4 mg/L, meropenem 0.25-2 mg/L, vancomycin 0.5-1 mg/L, and linezolid 0.25-4 mg/L.

For disk diffusion and Etest, preparation of inocula, inoculation and incubation of plates were performed according to EUCAST disk diffusion methodology using a McFarland 0.5 inoculum on Mueller-Hinton (MH) agar plates with 5% defibrinated horse blood and 20 mg/L β-NAD (MH-F plates) and incubation at 35 °C in 5% CO_2_ for 16-20 hours. If poor growth was observed after 16-20 h incubation, isolates were re-incubated and inhibition zones and MICs read after a total of 40-44 h incubation. Zone edges were read as the point showing no growth viewed with the naked eye from the front of the plate with the lid removed and with reflected light. BMD (Sensititre) was performed using a 5 x 10^5^ CFU/mL inoculum prepared in MH-F broth [19] added to Sensititre STP6F BMD plates and sealed before incubation at 35 °C in ambient air for 16-20 h before reading. As for disk diffusion and Etest, the BMD plates were re-incubated and read after a total of 40-44 h if growth was poor after 16-20 h incubation.

Simultaneous with the clinical isolates, the QC strain *Streptococcus pneumoniae* ATCC 49619 was tested for susceptibility to the six examined antimicrobial agents using the three susceptibility testing methods.

## 3 RESULTS

On MH-F agar, confluent lawn of growth was observed after 16-20 h incubation for *A. urinae* and *A. sanguinicola* isolates. By disk diffusion, Etest, and BMD determinations, 14 of the *A. urinae* isolates had weak growth after 16-20 h incubation and needed prolonged incubation (40-44 h).

Antimicrobial susceptibility test results (disk diffusion zone diameters, MIC_50_, MIC_90_, and MIC ranges) with the three used methods are given in (Tables **1** and **2**), for *A. urinae* and *A. sanguinicola* isolates, respectively. Zone histograms of disk diffusion against corresponding BMD MICs are visualized using different colors for each MIC in the supplementary material. Normal MIC distributions were obtained for both methods (data not shown). Using the EUCAST recommendations, Etest and BMD MIC values were obtained for 76-81 *A. urinae* strains and for 38-39 *A. sanguinicola* strains.

For the wild-type population of *A. urinae* isolates, the range of zone diameters differed among examined antimicrobial agents from 6 mm (vancomycin) to 14 mm (cefotaxime). For *A. sanguinicola*, wild-type populations ranged from 5 mm (vancomycin) to 11 mm (meropenem).

## 4 DISCUSSION

CLSI (August 2016) and EUCAST (January 2017) have currently established species-specific breakpoints for *A. urinae* and *A. sanguinicola* and before the availability of such data, MICs needed to be interpreted using breakpoints established for related bacteria as viridans group streptococci.

The EUCAST standardized disk diffusion method for fastidious microorganisms uses Mueller-Hinton agar supplemented with 5% defibrinated horse blood and 20 mg/L β-NAD (MH-F), and a standard inoculum of McFarland 0.5 for obtaining sufficient demarcating growth following 16-20 h incubation in ambient air supplemented with 5% CO_2_. In a study by Humphries & Hindler [9] they demonstrated good growth of 128 *A. urinae* isolates when using cation-adjusted Mueller-Hinton (MH) broth (CAMHB) supplemented with 2.5% to 5% lysed horse blood (LHB), which is the CLSI recommended standard medium (CAMHB-LHB). In the present study, growth of *A. urinae* isolates were weaker as compared to *A. sanguinicola* isolates and prolonged incubation were needed for 14 of the *A. urinae* isolates to obtain readable growth.

In Table **3** the recommended MIC breakpoints by EUCAST and CLSI in addition to EUCAST recommended zone diameter breakpoints are given for all of the examined antibiotics in this study. The EUCAST recommendations cover *A. urinae* and *A. sanguinicola* and the CLSI recommendations additionally include *Aerococcus viridans*. The inhibition zone diameters and MICs obtained were within CLSI and EUCAST recommendations for being susceptible, except for one *A. urinae* strain using cefotaxime [15, 16].

Broth microdilution and Etest MICs from this study corresponded to MIC values using BMD, agar dilution, and gradient tests as reported in a review from Rasmussen M [1]. Rasmussen M [1] summarized published antibiotic susceptibility testing results of *A. urinae* and *A. sanguinicola* isolates using EUCAST breakpoints for viridans group streptococci for 13 antibiotics for between 58 to 342 *A. urinae* isolates and between 8 to 65 *A. sanguinicola* isolates. The antibiotic susceptibilities varied slightly between the two species. Both *Aerococcus* species were susceptible to β-lactam antibiotics with modal MICs for penicillin based on 308 *A. urinae* and 65 *A. sanguinicola* isolates in the MIC range of 0.03-0.06 mg/L [1]. In the present study, penicillin susceptibilities were in agreement with these findings. Penicillin MICs for *A. sanguinicola* isolates were in general one dilution higher than MICs for *A. urinae*. When comparing MICs and zone diameters, all isolates were susceptible using the EUCAST breakpoints. The MICs for cephalosporins, such as cefotaxime and ceftriaxone, have been reported close to the upper limit for susceptibility, but still often within the range of being susceptible, using the CLSI breakpoints for streptococci [1]. In the study of Humphries & Hindler [9] which was based on 128 *A. urinae* isolates, all but one isolate tested were susceptible to meropenem (MIC ≤ 0.5 mg/L), with a modal MIC of ≤ 0.015 mg/L. The sole meropenem-nonsusceptible isolate was also reproducibly reduced susceptible to ceftriaxone (4 mg/L) and cefotaxime (2 mg/L), but susceptible to penicillin (0.06 mg/L). One of the *A. urinae* isolates in this study demonstrated an Etest MIC value one dilution above the CLSI breakpoint for cefotaxime, thereby being intermediate susceptible, but zone diameter and BMD (Sensititre) MIC determinations indicated susceptibility. For meropenem, MIC ranges were observed as ≤ 0.25 mg/L for both *Aerococcus* species.

Both species being capable of causing urinary tract infections, septicemia, and though seldom infective endocarditis makes it favorable that isolates exhibited no resistance to penicillin. Two earlier studies have additionally indicated that there is a synergistic killing effect when combining penicillin and an aminoglycoside [10, 20], though a new larger study could only demonstrate such synergy in a minority of isolates [2]. This favorable susceptibility pattern is important as both organisms essentially are uropathogens, that may be resistant to more of the otherwise empirically used antibiotics for treating urinary tract infections (nalidixic acid, trimethoprim, sulfonamides, and mecillinam), though, the degree of resistance and methods for their detection are being debated [5, 7, 9].

Aerococci, as other Gram-positive cocci, except the inborn resistant pediococci, have modal MICs for vancomycin of 1 mg/L or below. This has been reported for at least 308 isolates of *A. urinae* and 23 isolates of *A. sanguinicola* [1] and makes vancomycin treatment of invasive infections as septicemia and infective endocarditis an attractive alternative in the penicillin allergic patient, eventually in combination with gentamicin. Except for two *A. urinae* isolates (Etest MIC 2 mg/L), results from the current study demonstrated vancomycin MICs for *A. urinae* and *A. sanguinicola* isolates ≤ 1 mg/L, which were in line with vancomycin MICs as reviewed by Rasmussen M [1] and susceptible according to EUCAST and CLSI breakpoints.

Our isolates of both species were susceptible to linezolid using CLSI breakpoint and susceptible to rifampicin (Etest testing only) using EUCAST breakpoints. Humphries & Hindler [9] also found isolates of both species linezolid susceptible, except two isolates with linezolid MICs of 4 and 8 mg/L. Sierra-Hoffman *et al*. [21, 22] examined 56 *A. urinae* isolates and Facklam *et al.* [11] 15 isolates of *A. sanguinicola* that were found susceptible to rifampicin, an antibiotic with a possible role in combination antibiotic therapy in severe infective endocarditis cases.

Some limitations of the study are the number of strains that were included in the study and the limited geographic distribution by the clinical strains only originating from Denmark, Switzerland, Spain, and the CCUG culture collection of Gothenburg (Sweden). Not all of the clinical strains were having sufficient growth by following the EUCAST and CLSI recommended incubation conditions, whereof no MIC values were stated for some of the strains. Another limitation by using a commercial BMD system is that only manufacturer selected antibiotic concentrations were tested and a MIC reading at the lowest MIC concentration can only identify the lowest value without identification of an exact MIC value. In a daily usage, the commercial BMD system is easy to use, reduces the hands-on time during preparation of the plates and less time consuming.

## CONCLUSION

In conclusion, *A. urinae* and *A. sanguinicola* are emerging pathogens, primarily uropathogens, but have also been etiologic agents of bacteremia/septicemia and infective endocarditis; the latter though seldom well documented for *A. urinae*. Suggestions on breakpoints for most relevant antibiotics were just recently released by CLSI and EUCAST. The data obtained in this study has been integrated as part of the background data for the EUCAST clinical breakpoint recommendations.

Data for six antimicrobial agents using disk diffusion, Etest, and BMD were obtained for 120 *A. urinae* and *A. sanguinicola* isolates. MIC values obtained by Etest and BMD were in accordance within ± one dilution. All isolates were found susceptible to the six antimicrobial agents of penicillin, cefotaxime, meropenem, vancomycin, linezolid, and rifampicin.

## Figures and Tables

**Table 1:** MICs and zone diameters for *A. urinae* isolates when tested by EUCAST standardized disk diffusion method, Etest, and broth microdilution (Sensititre).

***A. urinae***	**Disk diffusion (wildtype)**	**Etest**														Broth microdilution *		
		No. of isolates with MIC (mg/L)											MIC (mg/L)			MIC (mg/L)		
**Antibiotic**	mm	<0.002	0.004	0.008	0.016	0.032	0.064	0.125	0.25	0.5	1	2	MIC_50_	MIC_90_	Range	MIC_50_	MIC_90_	Range
Benzylpenicillin	29-40			8	31	28	10	1					0.032	0.064	0.008-0.125	≤0.03	0.06	≤0.03-0.06
Cefotaxime	24-38				1	5	20	22	18	8	3	1	0.125	0.5	0.016-2	0.25	1	≤0.125-2
Meropenem	35-47			5	19	29	21	2	3				0.032	0.064	0.008-0.25	≤0.25	≤0.25	≤0.25
Vancomycin	19-25							1	1	36	40	2	1	1	0.125-2	≤0.5	≤0.5	≤0.5
Linezolid	27-35								5	10	35	28	1	2	0.25-2	1	1	≤0.25-2
Rifampicin	34-46	5	11	27	26	5	1	1					0.008	0.016	<0.002-0.125	NR	NR	NR

* Susceptibility recommendation method: Medium: Mueller-Hinton (MH) plates with 5% defibrinated horse blood and 20 mg/L β-NAD (MH-F). Inoculum: McFarland 0.5. Incubation: 5% CO_2_, 35±1 °C, 18±2h. Isolates with insufficient growth after 16-20 h incubation were re-incubated immediately and inhibition zones and MICs read after a total of 40-44 h incubation. Reading: Read zone edges as the point showing no growth viewed from the front of the plate with the lid removed and with reflected light.

**Table 2:** MICs and zone diameters for *A. sanguinicola* isolates when tested by EUCAST standardized disk diffusion method, Etest, and broth microdilution (Sensititre).

***A. sanguinicola***	**Disk diffusion (wildtype)**	**Etest**														Broth microdilution *		
		No. of isolates with MIC (mg/L)											MIC (mg/L)			MIC (mg/L)		
**Antibiotic**	mm	<0.002	0.004	0.008	0.016	0.032	0.064	0.125	0.25	0.5	1	2	MIC_50_	MIC_90_	Range	MIC_50_	MIC_90_	Range
Benzylpenicillin	25-34				1	15	20	2					0.064	0.064	0.016-0.125	≤0.03	0.06	≤0.03-0.06
Cefotaxime	24-34						1	11	12	11	3		0.25	0.5	0.064-1	0.25	1	≤0.125-1
Meropenem	34-45					9	23	7					0.064	0.125	0.032-0.125	≤0.25	≤0.25	≤0.25
Vancomycin	17-22								9	26	4		0.5	1	0.25-1	≤0.5	≤0.5	≤0.5-1
Linezolid	27-35									10	25	4	1	2	0.5-2	1	1	≤0.5-1
Rifampicin	28-35			1	2	12	18	5					0.064	0.125	0.008-0.125	NR	NR	NR

* Susceptibility recommendation method: Medium: Mueller-Hinton (MH) plates with 5% defibrinated horse blood and 20 mg/L β-NAD (MH-F). Inoculum: McFarland 0.5. Incubation: 5% CO_2_, 35±1 °C, 18±2h. Isolates with insufficient growth after 16-20 h incubation were re-incubated immediately and inhibition zones and MICs read after a total of 40-44 h incubation. Reading: Read zone edges as the point showing no growth viewed from the front of the plate with the lid removed and with reflected light.

**Table 3:** Recommendations given by EUCAST and CLSI for the six antibiotics examined in the present study.

**Guideline**	**EUCAST MIC breakpoint (mg/L)**		EUCAST Zone diameter breakpoint (mm) *		CLSI interpretive criteria MIC (mg/L) **		
**Antibiotic**	S ≤	R >	S ≥	R <	S ≤	I	R ≥
Benzylpenicillin^1^ / penicillin^2^	0.125	0.125	21	21	0.12	0.25-2	4
Cefotaxime	NR	NR	NR	NR	1	2	4
Meropenem	0.25	0.25	31	31	0.5	NR	NR
Vancomycin	1	1	16	16	1	NR	NR
Linezolid	NR	NR	NR	NR	2	NR	NR
Rifampicin	0.125	0.125	25	25	NR	NR	NR

S, Susceptible. I, Intermediate. R, Resistant. NR, No recommendation. ^1^ Benzylpenicillin for EUCAST testing. ^2^ Penicillin for CLSI testing. Quality control strain *Streptococcus pneumoniae* ATCC 49619 for both EUCAST and CLSI. * Susceptibility recommendation method: Medium: Mueller-Hinton (MH) plates with 5% defibrinated horse blood and 20 mg/L β-NAD (MH-F). Inoculum: McFarland 0.5. Incubation: 5% CO_2_, 35±1 °C, 18±2h. Isolates with insufficient growth after 16-20 h incubation were re-incubated immediately and inhibition zones and MICs read after a total of 40-44 h incubation. Reading: Read zone edges as the point showing no growth viewed from the front of the plate with the lid removed and with reflected light. ** Susceptibility recommendation method: Medium: Cation-adjusted Mueller-Hinton broth (CAMHB) supplemented with 2.5% lysed horse blood (CAMHB-LHB). Inoculum: Direct colony suspension, equivalent to a 0.5 McFarland standard. Incubation: 35 °C, 5% CO_2_, 20-24 h.

## References

[1] Rasmussen M. (2016). Aerococcus: An increasingly acknowledged human pathogen.. Clin. Microbiol. Infect..

[2] Sunnerhagen T., Nilson B., Olaison L., Rasmussen M. (2016). Clinical and microbiological features of infective endocarditis caused by aerococci.. Infection.

[3] Shelton-Dodge K., Vetter E.A., Kohner P.C., Nyre L.M., Patel R. (2011). Clinical significance and antimicrobial susceptibilities of *Aerococcus sanguinicola* and *Aerococcus urinae*.. Diagn. Microbiol. Infect. Dis..

[4] Lupo A., Guilarte Y.N., Droz S., Hirzel C., Furrer H., Endimiani A. (2014). *In vitro* activity of clinically implemented β-lactams against *Aerococcus urinae*: Presence of non-susceptible isolates in Switzerland.. New Microbiol..

[5] Christensen J.J., Kilian M., Fussing V., Andresen K., Blom J., Korner B., Steigerwalt A.G. (2005). Aerococcus urinae: Polyphasic characterization of the species.. APMIS.

[6] Senneby E., Göransson L., Weiber S., Rasmussen M. (2016). A population-based study of aerococcal bacteraemia in the MALDI-TOF MS-era.. Eur. J. Clin. Microbiol. Infect. Dis..

[7] Cattoir V., Kobal A., Legrand P. (2010). *Aerococcus urinae* and *Aerococcus sanguinicola*, two frequently misidentified uropathogens.. Scand. J. Infect. Dis..

[8] Christensen J.J., Korner B., Casals J.B., Pringler N. (1996). Aerococcus-like organisms: Use of antibiograms for diagnostic and taxonomic purposes.. J. Antimicrob. Chemother..

[9] Humphries R.M., Hindler J.A. (2014). *In vitro* antimicrobial susceptibility of *Aerococcus urinae*.. J. Clin. Microbiol..

[10] Skov R., Christensen J.J., Korner B., Frimodt-Møller N., Espersen F. (2001). *In vitro* antimicrobial susceptibility of *Aerococcus urinae* to 14 antibiotics, and time-kill curves for penicillin, gentamicin and vancomycin.. J. Antimicrob. Chemother..

[11] Facklam R., Lovgren M., Shewmaker P.L., Tyrrell G. (2003). Phenotypic description and antimicrobial susceptibilities of *Aerococcus sanguinicola* isolates from human clinical samples.. J. Clin. Microbiol..

[12] Ibler K., Truberg JK., Ostergaard C., Sönksen U.W., Bruun B., Schønheyder H.C., Kemp M., Dargis R., Andresen K., Christensen J.J. (2008). Six cases of *Aerococcus sanguinicola* infection: Clinical relevance and bacterial identification.. Scand. J. Infect. Dis..

[13] Senneby E., Petersson A.C., Rasmussen M. (2015). Epidemiology and antibiotic susceptibility of aerococci in urinary cultures.. Diagn. Microbiol. Infect. Dis..

[14] Senneby E., Eriksson B., Fagerholm E., Rasmussen M. (2014). Bacteremia with *Aerococcus sanguinicola*: Case series with characterization of virulence properties.. Open Forum Infect. Dis..

[15] Clinical and Laboratory Standards Institute (2016). Methods for antimicrobial dilution and disk susceptibility testing of infrequently isolated or fastidious bacteria..

[16] The European Committee on Antimicrobial Susceptibility Testing (2017). Breakpoint tables for interpretation of MICs and zone diameters..

[17] The European Committee on Antimicrobial Susceptibility Testing (2017). EUCAST Disk Diffusion Test Manual.

[18] International Standards Organisation (1996). Reference method for testing the *in vitro* activity of antimicrobial agents against rapidly growing aerobic bacteria involved in infectious diseases..

[19] The European Committee on Antimicrobial Susceptibility Testing (5.0, 2017). Media preparation for EUCAST disk diffusion testing and for determination of MIC values by the broth microdilution method..

[20] Zbinden R., Santanam P., Hunziker L., Leuzinger B., von Graevenitz A. (1999). Endocarditis due to *Aerococcus urinae*: Diagnostic tests, fatty acid composition and killing kinetics.. Infection.

[21] Humphries R.M., Lee C., Hindler J.A. (2011). *Aerococcus urinae* and trimethoprim-sulfamethoxazole.. J. Clin. Microbiol..

[22] Sierra-Hoffman M., Watkins K., Jinadatha C., Fader R., Carpenter J.L. (2005). Clinical significance of *Aerococcus urinae*: A retrospective review.. Diagn. Microbiol. Infect. Dis..

